# Fractalkine Signaling Attenuates Perivascular Clustering of Microglia and Fibrinogen Leakage during Systemic Inflammation in Mouse Models of Diabetic Retinopathy

**DOI:** 10.3389/fncel.2016.00303

**Published:** 2017-01-10

**Authors:** Andrew S. Mendiola, Rolando Garza, Sandra M. Cardona, Shannon A. Mythen, Sergio A. Lira, Katerina Akassoglou, Astrid E. Cardona

**Affiliations:** ^1^Department of Biology and South Texas Center for Emerging Infectious Diseases, The University of Texas at San AntonioSan Antonio, TX, USA; ^2^Immunology Institute, Icahn School of Medicine at Mount SinaiNew York, NY, USA; ^3^Gladstone Institute of Neurological Disease, University of California, San FranciscoSan Francisco, CA, USA; ^4^Department of Neurology, University of California, San FranciscoSan Francisco, CA, USA

**Keywords:** microglia, fractalkine, CX3CR1, diabetic retinopathy, retina, fibrinogen

## Abstract

Fractalkine (FKN) is a chemokine expressed constitutively by healthy neurons and signals to microglia upon interaction with the FKN receptor, CX3CR1. Signaling between FKN and CX3CR1 transduces inhibitory signals that ameliorate microglial activation and proinflammatory cytokine release in neuroinflammatory conditions. The aim of this study is to determine the mechanisms associated with microglial activation and vascular leakage during diabetic retinopathy (DR) and under conditions of low-level endotoxemia, common in diabetic patients. Utilizing the Ins2^Akita^ strain (Akita), a mouse model of type 1 diabetes, our results show that leakage of the blood-protein fibrin(ogen) into the retina occurs as a result of chronic (4 months) but not acute (1.5 months) hyperglycemia. Conversely, inducing endotoxin-mediated systemic inflammation during acute diabetes resulted in fibrinogen deposition in the retina, a phenotype that was exacerbated in mice lacking CX3CR1 signaling. Systemic inflammation in *Cx3cr1^−/−^* mice led to robust perivascular clustering of proliferating microglia in areas of fibrinogen extravasation, and induced IL-1β expression in microglia and astrocytes. Lastly, we determined a protective effect of modulating FKN/CX3CR1 signaling in the diabetic retina. We show that intravitreal (iv) administration of recombinant FKN into diabetic FKN-KO mice, reduced fibrinogen deposition and perivascular clustering of microglia in the retina during systemic inflammation. These data suggest that dysregulated microglial activation via loss of FKN/CX3CR1 signaling disrupts the vascular integrity in retina during systemic inflammation.

## Introduction

Endotoxin levels—specifically bacterial lipopolysaccharide (LPS)—are found elevated in the sera of diabetic patients contributing to disease incidence by upregulating IL-1β, IL-6, TNF-α, C-reactive protein, plasminogen activator inhibitor type 1 (PAI-1) and fibrinogen (Cani et al., 2007; Lassenius et al., 2011; Pussinen et al., 2011). Apart from being a risk factor for infectious diseases and atherosclerosis (Shah and Hux, 2003), hyperglycemia also poses an increased risk of thrombotic events, evident by activation of the coagulation system and inhibition of the fibrinolytic system (Carr, 2001) in diabetic patients. Fibrinogen also known as coagulation factor I, is abundant in the blood and functions in the coagulation cascade creating polymers of fibrin. Fibrin clots form in response to injuries to any part of the vascular system. In the nervous tissue, fibrinogen is deposited as insoluble fibrin once the blood-brain barrier is compromised (Davalos et al., 2012). It has been shown that soluble fibrinogen in the bloodstream is not proinflammatory, but fibrin formation as a result of the activation of the coagulation cascade exposes fibrin cryptic epitopes that lead to activation of innate immunity (Bardehle et al., 2015). While a prominent role of fibrinogen exists in mediating cellular activation and tissue damage in models of neuroinflammation (Bardehle et al., 2015), less is known in diabetic retinopathy (DR).

DR is a progressive retinal disease and leading cause of blindness in diabetics. Growing evidence suggests neuroinflammation plays an early role in mediating neuronal and vascular pathology in DR (Zheng et al., 2007; Tang and Kern, 2011; Li et al., 2012). Microglia, which are normally engaged with neurons and taking part in synaptic maintenance and clearance of cellular debris (Sierra et al., 2010; Paolicelli et al., 2011), are now recognized as being more than just intrinsic “bystanders”, but immune cells with key roles in injury and repair. In particular, during diabetes, inhibition of microglial activation via administration of minocycline attenuates both neuronal (Krady et al., 2005) and vascular (Vincent and Mohr, 2007) degeneration in the retina by dampening the proinflammatory response. Several studies support the involvement of microglia-mediated inflammation in DR pathogenesis. However, the impact of systemic inflammation in microglia phenotype in a diabetic host is enigmatic. Therefore, understanding the mechanisms that lead to retinal microglia activation may provide unique therapeutic target to regulate inflammatory reactions and ameliorate pathology during retinopathy.

In the central nervous system (CNS), neurons constitutively express Fractalkine (FKN), which binds to CX3CR1 on microglia (Bazan et al., 1997; Imai et al., 1997; Combadiere et al., 1998; Rossi et al., 1998; Jung et al., 2000; Cook et al., 2001). Disruption of CX3CR1 signaling influences microglial physiology in health and disease (Limatola and Ransohoff, 2014). For example, microglia in CX3CR1-KO mice are proinflammatory prone and contribute to exacerbated neurotoxicity in several models of neurodegeneration (Cardona et al., 2006) and also in DR (Cardona et al., 2015). Moreover, it was recently reported that CX3CR1-deficiency accelerates vascular degeneration following chronic diabetes (Beli et al., 2016). These data suggest a relationship between CX3CR1 and maintenance of the vascular integrity in the diabetic retina. Yet, the contributions of dysregulated microglial activation on retinal vascular damage during episodes of acute systemic inflammation remain unknown.

Here, we sought to determine how CX3CR1 signaling modulates microglial-vasculature responses during systemic inflammation in the acute diabetic retina. We report that following chronic (16 weeks), but not acute (6 weeks) diabetes, fibrinogen leakage was evident in the retina irrespective of the CX3CR1 genotype. In contrast, systemic inflammation induced fibrinogen leakage in the acute diabetic retina, and this effect was exacerbated in diabetic CX3CR1-KO mice and correlated with perivascular clustering of activated microglia. Interestingly, systemic LPS lead to robust retinal vascular leakage in nondiabetic CX3CR1-KO but not CX3CR1-HET mice. Lastly, we show that intravitreal (iv) administration of recombinant FKN into diabetic FKN-KO mice, reduced fibrinogen deposition and perivascular clustering of microglia in the retina during systemic inflammation.

## Materials and Methods

### Mouse Models

C57BL/6, Cx3cr1^gfp/gfp^ (CX3CR1-KO), and Insulin2^Akita^ (Akita) mice were purchased from The Jackson Laboratory and breeding pairs of *Cx3cl1^−/−^* (FKN-KO) mice (Cook et al., 2001) were obtained from Sergio Lira (Icahn School of Medicine at Mount Sinai). All mice were in-bred and maintained at the animal facilities at the University of Texas at San Antonio. In this study, CX3CR1-KO mice were crossed with the Akita type 1 diabetic strain to generate Akita CX3CR1-KO (Akita-KO), Akita CX3CR1-HET (*Cx3cr1*^+/gfp^; Akita-HET) and Akita CX3CR1-WT (*Cx3cr1*^+/+^; Akita-WT) mice as previously described (Cardona et al., 2015). Aged-matched nondiabetic and diabetic animals of their respective *Cx3cr1* genotype served as controls. Akita male mice become hyperglycemic by 4 weeks old, and animals were maintained for 6 weeks (10 weeks old) or 16 weeks (20 weeks old) thereafter to study acute and chronic diabetes, respectively. Blood glucose was measured using the Precision Xtra Kit via check puncture and animals were deemed diabetic when blood glucose levels were >400 mg/dL at time of experiment. This study was carried out in accordance with National Institutes of Health guidelines and approved by the UTSA Institutional Animal Care and Use Committee.

### Antibodies and Reagents

Rabbit anti-ionized calcium binding adaptor molecule-1 (IBA1; Wako; 1:4000), rabbit anti-fibrinogen (Clone: A0080; Dako; 1:500), rabbit anti-proliferating cell nuclear antigen (PCNA, Clone: C19; Abcam; 1:500), rat anti-nitric oxide synthase (NOS2; Clone: CXNFT; Ebioscience: 1:500) rat anti-pecam-1/CD31 (Clone: MEC 13.3; BD Pharmingen: 1:500), Rat anti-glial fibrillary acidic protein (GFAP, Clone: 2.2B10, Invitrogen; 1:4000), and biotinylated-isolectin B4 (IB4; 1:1000), and avidin-biotin peroxidase complex were acquired from Vector Labs. Species-specific secondary antibodies Cy3-goat anti-rabbit, Cy5-donkey anti-rat, biotinylated-goat anti-rabbit; biotinylated-goat anti-mouse; Cy3- and Cy5-streptavidin conjugated antibodies were purchased from Jackson Laboratories. Streptozotocin (STZ) and LPS from *E. coli*, serotype 055:B5 were obtained from Sigma-Aldrich.

### Induction of Systemic Inflammation via Lipopolysaccharide Injections

Acute endotoxemia (systemic inflammation) was established as previously described (Cardona et al., 2006; Chen et al., 2012). Briefly, acute diabetic and aged-matched nondiabetic male mice were injected intraperitoneally (i.p.) with LPS (20 μg/100 μL PBS/mouse/day; 1mg/kg of body weight) for four-consecutive days, or mock injected with an equal volume of sterile PBS. Aged-matched nondiabetic and diabetic animals of their respective *Cx3cr1* genotype served as controls for LPS-treated animals. Mice were then sacrificed 4 h after the final LPS injection for analysis as described below. In a selected experiment only two doses of LPS were used to determine earlier effects of systemically-induced inflammation on retinal fibrinogen leakage.

### Immunofluorescence, Confocal Microscopy and Image Analysis

Mice were transcardially perfused with cold saline buffer followed by cold fixative (4% paraformaldehyde; PFA). For retinal isolation, eyes were enucleated and postfixed in 4% PFA overnight, retinas were then isolated free from sclera/retinal pigmented epithelium and the resulting retinal flat mounts were used for immunohistochemical staining as previously described (Cardona et al., 2015). In brief, retinal tissues were incubated for 4 h at 4°C with 1% Triton-X 100 in 10% goat serum to permeablize tissue and block nonspecific secondary antibody staining, respectively. Next, primary antibodies were incubated in the same buffer overnight at 4°C followed by rigorous washes, and then tissues were incubated with species-specific secondary antibodies to visualize proteins of interest. Confocal microscopy was done using a Zeiss LSM 510 microscope and 3D compositions of confocal images were generated in Imaris software v7.2 (Bitplane). For each different immunohistochemical stain the entire flat mount of the retina was assessed, and a minimum of three images per retina per mouse were obtained and further analyzed. To quantify fibrinogen, microglial and astrocyte immunoreactivity in confocal images, raw images were uploaded to ImageJ (NIH), converted to 8-bit grayscale, and then a global automatic threshold was applied to each image. Fibrinogen, microglia and astrocytic signal per image were measured and expressed as immunoreactivity percent or percent area of entire image. For microglial cell counts, cells were manually counted in 40× images. Data were normalized to *X*, *Y* and *Z* coordinates (i.e., 210 μm × 210 μm × 15 μm) to account for changes in confocal *z-stack* thickness and expressed as microglia per mm^3^. To assess changes in microglial morphology, the transformation index (TI) was determined in microglial cells as previously described (Cardona et al., 2015). Briefly, 40× confocal images were uploaded to ImageJ and the cellular perimeter and area were obtained and used in the equation: TI = Perimeter^2^/4*Π*Area. A circular shape has a TI equal to 1 and when used to evaluate microglial morphology, cells with ameboid shape have values closer to 1 whereas ramified cells have much larger values. Lastly, the Imaris Coloc tool was used to quantify the colocalization between two fluorochromes in an image. The two channels of interest were selected and an automatic threshold was performed on both channels. Imaris Coloc volume statistics was used to present data as the number of total colocalized voxels between two channels.

### Quantitative PCR

RNA was extracted from isolated retinas following the Trizol method (Invitrogen), and quality control parameters assessed as previously described (Jung et al., 2000; Cardona et al., 2015). 500 ng of total RNA was reverse-transcribed to cDNA using the High-Capacity cDNA Reverse Transcription Kit (Applied Biosystems). Samples were run in triplicate in 10 μL PCR reactions containing 1X SYBR Green PCR Master Mix (Applied Biosystems), 250 nM forward and reserve primers, and 20 ng cDNA template. qPCR reactions were run in a 384-well plate format using a 7900 HT Fast Real-Time PCR System (Applied Biosystems). Results were analyzed by the comparative Ct method as previously described (Cardona et al., 2015). Data are expressed as 2^-∆∆Ct^ for the experimental gene of interest normalized to two separate housekeeping genes, *Rn18s* (18s) and *Actb* (β-actin), and presented as the average fold change relative to naive genotype-specific controls. The following primers were used: *Il1b* (Accession number: NM_008361.3): Forward (Fwd) 5′-GTGTGGATCCAAAGCAATAC-3′, Reverse (Rev) 5′-GTCTGCTCATTCATGACAAG-3′; *Nos2* (Accession number: NM_010927.4): Fwd 5′-GGCAGCCTGTGAGACCTTTG-3′, Rev 5′-TGCATTGGAAGTGAAGCGTTT-3′; *18s* (Accession number: NR_003278.3): Fwd 5′-CGGCTACCACATCCAAGGAA-3′, Rev 5′-GTCGGAAATACCGCGGTC-3′; *β-actin* (Accession number: NM_007393.3): Fwd 5′-CTCTGGCTCCTAGCACCATGAAGA-3′, Rev 5′-GTAAAACGCAGCTCAGTAACAGTCCG-3′.

### STZ-Induced Diabetes and Intravitreal Administration of Recombinant Fractalkine

For FKN treatment experiments, FKN-KO were rendered diabetic by administration of multiple-low doses of STZ as previously described (Krady et al., 2005). In brief, mice received daily i.p. injections of STZ (60 mg/kg/day) dissolved in citric acid buffer (50 mM; pH 4.5) for five-consecutive days. Blood glucose was monitored weekly after STZ injections, and mice were sacrificed following 1 month of hyperglycemia reflected by blood glucose levels >400 mg/dL. Citrate buffer injected mice were used as controls (data not shown). Acute (2 day LPS challenge) systemic inflammation was induced as described above in diabetic FKN-KO mice. Following the second LPS injection, mice received an iv injection of FKN or mock injection with sterile PBS. In brief, mice were anesthetized with 5% isoflurane in oxygen and injections were administered under a dissecting microscope with a 31G beveled Nanofil needle and syringe connected to a Micro 4^TM^ Microsyringe Pump Controller (World Precision Instruments). Prior to injections, a drop of 0.5% proparacaine hydrochloride ophthalmic solution (Akron) was applied to each eye as a topical anesthetic. One eye received an iv injection of recombinant mouse CX3CL1/ FKN chemokine domain (Leu22-Lys105; R&D Systems) infused at 200 nL/s for a total of 5 s (diluted in PBS; 30 ng/μL, 1 μL injections). The contralateral eye received a mock injection with an equal volume of sterile PBS. Following 24 h after the second LPS and iv injections, mice were sacrificed and their retinas analyzed.

### Statistical Analysis

Statistical tests’ performed include two-tailed unpaired Student’s *t* test when comparing two groups and one-way ANOVA followed by Tukey’s *post hoc* test for multiple comparisons. All analyses were conducted in GraphPad Prism v5.0 and a *P* value <0.05 was considered statistically significant.

## Results

### Fibrinogen Is Detected in Retinal Tissues of Diabetic Mice at Chronic but Not Acute Stages of Disease

**Figure 1:**
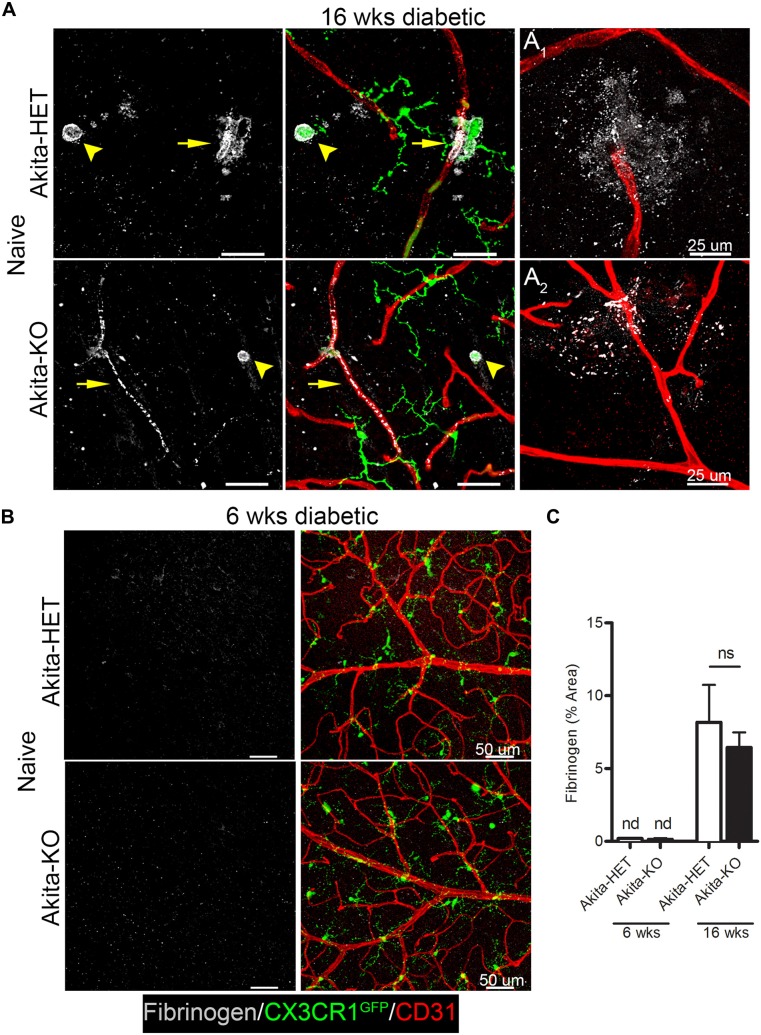
**Fibrinogen is detected in the retina at chronic stages of diabetes**. Vascular leakage in naïve diabetic mice (*Ins2^Akita^* strain) at chronic (16 weeks) and acute stages (6 weeks) of diabetes were assessed via detection of fibrinogen in the retina. **(A,B)** Confocal images revealed fibrinogen (white) immunoreactive areas in retinal flat mounts of Akita CX3CR1-HET (Akita-HET) and Akita CX3CR1-KO (Akita-KO) mice at **(A)** chronic but not at **(B)** acute stages of hyperglycemia. Moreover, fibrinogen colocalized to areas of activated microglia (CX3CR1-GFP; arrowhead) near blood vessels (CD31, red; arrows). Representative examples of fibrinogen extravasation in **(A_1_)** Akita-HET and **(A_2_)** Akita-KO retinas are shown. **(C)** Fibrinogen immunoreactive area was quantified in confocal images and expressed as mean ± SD (*n* = 4–5 mice per genotype) ns = not significant, nd = not detectable.

We identified vascular leakage by staining for the blood-protein fibrinogen in naïve Akita-HET and Akita-KO retinas at chronic (16 weeks) diabetes. Fibrinogen staining colocalized with morphologically-activated microglia (Figures 1A,C) in areas closely associated with blood vessels. In some cases, fibrinogen was found not only tightly associated with blood vessels but also leaking into the parenchyma of the retina (Figure 1A_1,2_). Fibrinogen deposition in retinal blood vessels of Akita mice was not evident in acute (6 weeks) stages of diabetes regardless of CX3CR1 genotype (Figures 1B,C; Table 1). However, perivascular accumulation of fibrinogen during chronic stages (16 weeks) of diabetes was apparent, although microglial infiltration was minimal. These data suggest that retinal blood vessels exposed to chronic hyperglycemia and neuroinflammation associated with DR (Roy et al., 2017) is required for larger plasma proteins, such as fibrinogen, to leak into the diabetic retina.

**Table 1:** **Fibrinogen leakage in retinas of naïve (non-lipopolysaccharide (LPS) treated) animals**.

Genotype	Duration of diabetes	Fibrinogen detected in retina (mice)	Perivascular clustering microglia
CX3CR1-HET	–	0/6	No
Akita-HET	6 weeks	0/4	No
Akita-HET	16 weeks	5/5	Limited
CX3CR1-KO	–	0/6	No
Akita-KO	6 weeks	0/4	No
Akita-KO	16 weeks	4/5	Limited

### Systemic Inflammation Accelerates Fibrinogen Leakage in the Acute Diabetic Retina and CX3CR1 Controlled the Extent of Perivascular Clustering of Microglia

**Figure 2:**
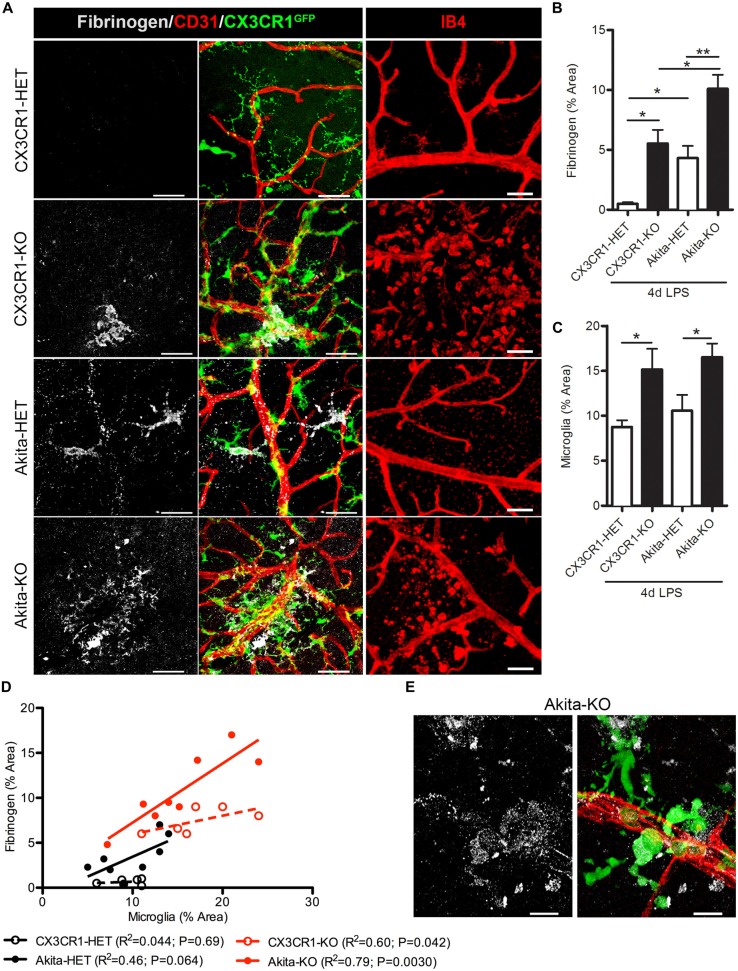
**Endotoxin-induced systemic inflammation triggers fibrinogen accumulation in the diabetic retina and correlates with perivascular microglia clustering in CX3CR1-KO mice. (A)** Retinal vascular leakage after systemic inflammation (4 days lipopolysaccharide (LPS)) was assessed by immunostaining for the blood-protein fibrinogen. Fibrinogen deposition (white) around retinal blood vessels (CD31; red) colocalized to microglial lesions (CX3CR1-GFP, green), except in nondiabetic CX3CR1-HET mice (Scale bars: 40 μm). Far right panels show evidence of isolectin B4+ (isolectin b4; IB4, red) cells around disrupted vascular staining in CX3CR1-KO mice (Scale bars: 50 μm). **(B)** Fibrinogen and **(C)** microglial CX3CR1-GFP immunoreactivity were quantified per confocal image and data presented as mean ± SEM (*n* = 4–7 mice per group). *P < 0.05 by ANOVA with Tukey’s HSD (See Table 2 for incidence). **(D)** Scatter plot analysis of fibrinogen % Area as a function of microglia % Area from data in panels **(B,C)**. **(E)** Representative high-power confocal image of LPS-treated Akita-KO retina immunostained for fibrinogen (white), microglia (green) and vasculature (CD31, red). Scale bars: 15 μm. ^**^P < 0.01.

Increase levels of plasma LPS, a major component of gram negative bacteria cell walls, the high prevalence of recurrent infections, and the associated increase of circulating pro-inflammatory cytokines in hyperglycemic patients (Cani et al., 2007; Lassenius et al., 2011; Pussinen et al., 2011) pose an important and unexplored intersection between systemic inflammation and DR progression. Immunohistochemical analyses revealed that fibrinogen was not detected in nondiabetic CX3CR1-HET mice challenged with 4 day LPS (Figures 2A,B; Table 2). Whereas, fibrinogen deposition was evident in close association to retinal blood vessels in nondiabetic CX3CR1-KO mice and colocalized to areas of perivascular clustering of microglia (Figure 2A). Furthermore, immunostaining showed perivascular IB4+ microglia/monocytes around aberrant vascular staining (Figure 2A); consistent with literature showing that microglia/myeloid cells upregulate IB4 upon activation (Chen et al., 2010). Interestingly, in acute diabetic mice (6 weeks), LPS had an augmented effect and resulted in vascular leakage evident with fibrinogen accumulation in the retinas of diabetic Akita mice, mainly in the Akita-KO retinas (Figures 2A,B). More specifically, fibrinogen staining in the retina was detected in 50% of LPS-treated Akita-HET mice, whereas fibrinogen deposition was present in 88% of LPS-treated Akita-KO animals (Table 2). Importantly, areas of fibrinogen immunoreactivity only correlated with microglial activation (immunoreactivity) in LPS-treated CX3CR1-KO and Akita-KO retinas (Figures 2C–E). Together, these experiments show that elevated systemic inflammation can accelerate breakdown of the blood-retinal barrier (BRB) as evidenced by fibrinogen extravasation. Additionally, these data suggest that CX3CR1 signaling may play a role in maintaining the BRB during insult, perhaps indirectly by modulating protective microglial responses in both nondiabetic and diabetic mice.

**Table 2:** **Fibrinogen leakage in retinas of LPS-treated animals**.

Genotype	Duration of diabetes	Treatment	Fibrinogen detected in retina (mice)	Perivascular clustering microglia
CX3CR1-HET	–	2 day LPS	0/3	No
CX3CR1-HET	–	4 day LPS	0/9	No
Akita-HET	6 weeks	4 day LPS	4/8	Limited
CX3CR1-KO	–	2 day LPS	3/3	Yes
CX3CR1-KO	–	4 day LPS	5/8	Yes
Akita-KO	6 weeks	4 day LPS	7/8	Yes
FKN-KO	–	2 day LPS	3/4	Yes
FKN-KO	–	4 day LPS	4/4	Yes
FKN-KO	4 weeks	2 day LPS + PBS (iv)*	4/4^#^	Yes
FKN-KO	4 weeks	2 day LPS + FKN (iv)*	4/4^#^	Yes

Data are presented as number/proportion of mice that score positive for fibrinogen deposition in the retina and in which the staining was closely associated to blood vessels, or visualized in the parenchyma of the neural retina. ^*^Intravitreal (iv) injection of either sterile PBS or recombinant fractalkine (FKN). ^#^A significant reduction in fibrinogen signal was observed following FKN treatment relative to PBS and data are shown in Figures 6H,I.

### Early Fibrinogen Deposition Correlates to the Microglial Clustering Phenotype Observed in CX3CR1-KO Mice

**Figure 3:**
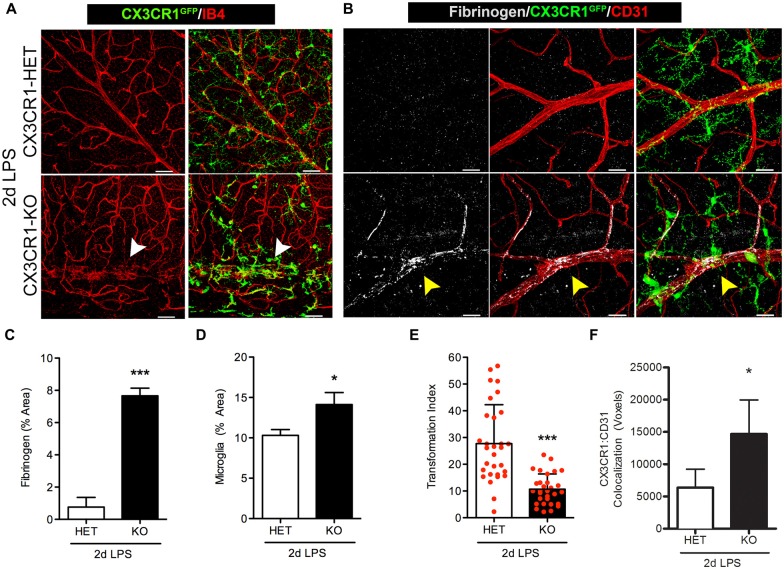
**Fibrinogen deposition and microglial activation is induced in *Cx3cr1*^**−/−**^ mice early after endotoxin-induced systemic inflammation**. Nondiabetic mice were challenged with LPS for two consecutive days at which **(A)** microglial activation (CX3CR1^GFP^) and cell clustering near vessels (IB4; red) was evident in CX3CR1-KO but not in CX3CR1-HET mice. Microglial clustering and abnormal vascular staining (white arrowheads) were associated with **(B)** fibrinogen (white) immunoreactivity in retinal blood vessels (CD31, red) of CX3CR1-KO mice only (yellow arrowhead). **(C)** Fibrinogen and **(D)** microglia immunoreactivity were compared among groups. **(E)** Transformation index (TI) was quantified per image and used to assess changes in microglial morphology in response to systemic LPS (20–30 individual microglial cells quantified per mouse; *n* = 3). **(F)** Microglia (CX3CR1-GFP) interaction with CD31+ blood vessels was assessed by measuring the colocalization (voxels) of GFP and CD31 fluorescence. Data in **(C–F)** are expressed as mean ± SD, *n* = 3. *P < 0.05, ***P < 0.001 by Student’s *t*-test. Scale bars: 50 μm.

We next assessed retinas for fibrinogen leakage at an earlier time point during systemic inflammation, 2 days after LPS administration. Since the 4 day LPS data shows a dramatic difference between the CX3CR1-KO and control groups, this experiment was designed to make sure that mice that express CX3CR1 do not show blood vessel abnormalities early after one or two doses of LPS that may have resolved by the fourth dose. As proof of concept we only performed these experiments in nondiabetic animals since fibrinogen leakage and perivascular clustering of microglia was evident in nondiabetic 4-day LPS-treated CX3CR1-KO mice. The pattern of the vascular staining in 2 days LPS-treated CX3CR1-KO mice was discontinuous and aggregated clumps of the endothelium were observed around perivascular microglial clusters (Figure 3A; white arrowheads). Furthermore, as observed in the 4-day challenge, 2 day LPS-treated CX3CR1-KO mice, showed increase perivascular deposition of fibrinogen (Figures 3B,C; yellow arrowhead), which coincided with increase microglial activation/immunoreactivity (Figure 3D). Conversely, minimal fibrinogen staining was detected histochemically in retinas of 2 day LPS-treated CX3CR1-HET mice (Figures 3B,C). The extent of morphological microglial activation (Figures 3A,B) was supported by assessment of TI, which accounts for cellular perimeter and area. The results confirmed that 2 day LPS challenge significantly altered the morphology of microglia in CX3CR1-KO relative to CX3CR1-HET mice (Figure 3E). CX3CR1-HET microglia appeared to have smaller cell bodies, long-ramified processes (Figures 3B,E), and a uniform distribution throughout the retina based on CX3CR1-GFP: CD31 colocalization analysis (Figure 3F). In contrast, CX3CR1-KO microglia displayed classic ameboid morphology evident of morphological activation and microglia were significantly more associated with the vasculature (Figure 3F). These data bolster the rationale that CX3CR1 controls the normal physiology of microglial during insult and that in absence of CX3CR1, low levels (1 mg LPS per kg animal) of acute (2 days) systemic inflammation is sufficient to make retinal blood vessels leaky.

### Clustering Phenotype of Morphologically-Activated CX3CR1-Deficient Microglia Correlates with Increased Numbers of Proliferating Cells in the Retina during Systemic Inflammation

**Figure 4:**
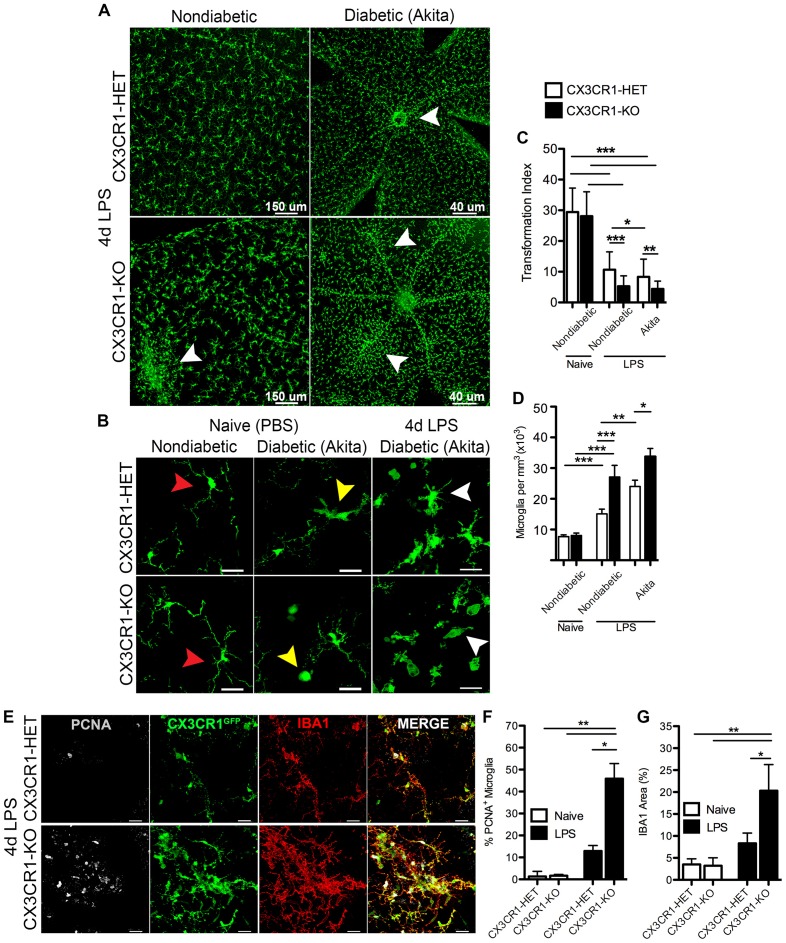
**Systemic inflammation promotes microgliosis and perivascular clustering of microglia in the retina of CX3CR1-deficient mice. (A)** Confocal images of microglia (CX3CR1-GFP, green) in retinal flat mounts following 4 d LPS challenge in nondiabetic CX3CR1-HET and CX3CR1–KO mice and diabetic Akita-HET and Akita-KO tissues. Robust microglial clustering near retinal blood vessels (white arrowheads) was observed in nondiabetic LPS-treated CX3CR1-KO but not in LPS-treated CX3CR1-HET mice. Perivascular clustering of microglia was exacerbated in LPS-treated Akita-KO compared to Akita-HET mice. **(B)** Images of higher magnification illustrating global microglia morphological activation in LPS-treated diabetic retinas (white arrowheads) in comparison to both naïve (PBS) nondiabetic (red arrowheads) and naïve diabetic retinal microglia (yellow arrowheads). **(C)** The TI was quantified in individual microglial cells to assess changes in morphology (*n* = 6 mice per group; 20–30 microglia quantified per mouse). Values closer to one represent cells with more circular and ameboid shape. **(D)** Microglial cell numbers were quantified in retinal tissues and normalized to the volume of each *z-stack* (*n* = 6–8 mice per group; three images per retina per mouse). **(E)** Retinal tissues of 4 day LPS-challenged nondiabetic CX3CR1-HET and CX3CR1-KO mice were stained with antibodies against proliferation cell nuclear antigen (PCNA, white) and ionized calcium binding adaptor molecule-1 (IBA1; red), which colocalized to CX3CR1-GFP reporter (green). Scale bars: 25 μm in **(B,E)**. **(F)** Image quantification of PCNA^+^ microglia (GFP+ and IBA1+) and **(G)** IBA1 immunoreactivity in LPS- or PBS-treated (naïve) mice (*n* = 3 mice per group; three images per mouse). Data are presented as mean ± SD in **(C,F,G)** and SEM in **(D)** *P < 0.05, **P < 0.01, ***P < 0.001 by ANOVA followed by Tukey’s HSD test.

We sought to investigate the role of CX3CR1 deficiency in microglia activation in response to systemic inflammation under physiological and diabetic states. Endotoxin-induced inflammation (4 day challenge) did not induce significant changes to the distribution of retinal microglia in CX3CR1-HET mice (Figure 4A). In contrast, 4 day LPS-treated CX3CR1-KO mice displayed robust microglial lesions or clusters around retinal blood vessels (Figure 4A, bottom left, white arrowhead). This unique clustering phenotype of microglia was observed primarily in the peripheral retina distal to the optic nerve head and was limited to one or two clusters per retina in nondiabetic CX3CR1-KO mice, whereas no microglial clustering was apparent in nondiabetic CX3CR1 wild type or heterozygous mice. In 4 day LPS-treated Akita-HET mice (Figure 4A, top right), microglial cells were grouped near blood vessels mostly at the optic nerve head (Figure 4A, top right; white arrowhead). However, in Akita-KO mice, systemic LPS had an exacerbated effect on microglial activation in the diabetic retina, represented by global perivascular clustering (Figure 4A, bottom right; white arrowheads). This phenotype was intensified in comparison to nondiabetic CX3CR1-KO mice and perivascular lesions of microglia were observed throughout the entire tissue involving multiple aggregations (Figure 4A, bottom right).

Images from naïve nondiabetic retinal tissues revealed classic ramified nonreactive microglial cells in both CX3CR1-HET and CX3CR1-KO mice (Figure 4B, red arrowheads). Furthermore, in agreement with our previous report (Cardona et al., 2015), acute diabetes was sufficient to activate microglia in naïve Akita-HET and Akita-KO retina—which exhibit morphological characteristics of reactive microglia including truncated cellular processes and ameboid morphology that is consistent with descriptions of a reactive/activated phenotype (Figure 4B, yellow arrowheads). Systemic inflammation (4 days) exacerbated the extent of microglial activation in both the nondiabetic (Figure 4A) and Akita-HET and Akita-KO retinas (Figure 4B, white arrowheads). The use of the TI to assess changes in microglial morphology confirmed that systemic LPS had a significant effect on microglial activation in both nondiabetic and diabetic groups relative to naïve nondiabetic controls (Figure 4C). However, the response to systemic inflammation was more prominent in CX3CR1-deficient mice than in CX3CR1-sufficient groups (Figure 4C). Interestingly, diabetes alone promoted a significant effect on microglial morphology as revealed by the data comparing CX3CR1-HET and Akita-HET mice (Figure 4C). Furthermore, image analysis showed that 4 day LPS treatment increased the number of microglial cell numbers in all groups, but similarly to the TI quantification, the change was more evident in CX3CR1-KO mice (Figure 4D).

To distinguish if these microglia foci were formed in response to cellular mobilization or due to microglial proliferation, retinal tissues were stained with the mitotic marker PCNA. This experiment was limited to nondiabetic animals since the clustering phenotype of microglia was observed in both nondiabetic and diabetic CX3CR1-KO mice. Following 4 days of LPS-treatment, retinal CX3CR1-KO microglia showed increased immunoreactivity for PCNA, which colocalized with the specific CX3CR1-GFP reporter protein and the microglial marker IBA1 (Figure 4E). CX3CR1-KO mice displayed a significant increase in the number of PCNA^+^ retinal microglia when compared to CX3CR1-HET mice (Figure 4F). Additionally, CX3CR1-KO microglia increased their expression of IBA1 by 2.2-fold in response to systemic LPS (Figure 4G). These data suggest that retinal microglia are highly sensitive to systemic inflammation, and the rapid response involves intense microglial reactivity and proliferation at targeted vascular sites where fibrinogen leakage was evident.

### In Response to Systemic Inflammation CX3CR1 Deficient Microglia Exhibit an Activated Phenotype Associated With Upregulation of the Proinflammatory Mediators IL-1β and iNOS

**Figure 5:**
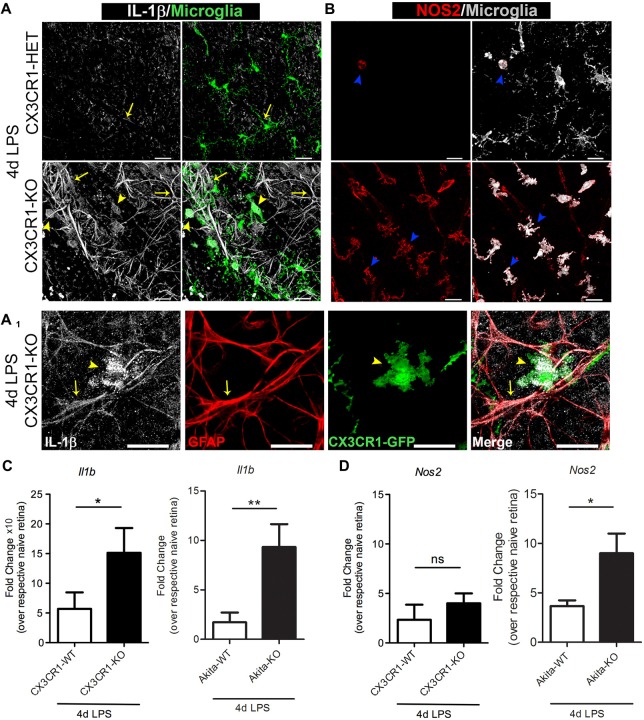
**Enhanced microglia activation in CX3CR1-KO retinas based on upregulation of IL-1β and NOS2. (A,B)** Confocal images of retinal flat mounts from nondiabetic 4 day LPS-treated CX3CR1-HET and CX3CR1-KO mice immunostained for **(A)** IL-1β (white), **(A_1_)** glial fibrillary acidic protein (GFAP; red) and **(B)** inducible nitric oxide synthase (iNOS or NOS2, red). Microglia visualized by virtue of the CX3CR1-GFP reporter are shown in green in **(A)** and pseudo-colored to white in **(B)**. IL-1β staining colocalized to both microglia (**A,A_1_**, yellow arrowheads), and astrocytes (**A,A_1_**; GFAP staining (red), yellow arrows) around blood vessels. iNOS^+^ activated microglia were abundant in retinas of LPS-treated CX3CR1-KO mice relative to CX3CR1-HET mice (**B**, blue arrowheads). Scale bars: 25 μm. **(C,D)** qRT-PCR analyses of **(C)** *Il1b* and **(D)** *Nos2* transcripts in whole retinal extracts are presented as mean fold change (from ddCT) over respective naïve genotype ± SD for *n* = 4 mice per group, *P < 0.05, **P < 0.01, ns = not significant, by ANOVA with Tukey’s HSD.

FKN signaling controls the proinflammatory phenotype of microglia in the brain during systemic inflammation and in animal models of amyotrophic lateral sclerosis (ALS), and Parkinson’s disease (PD; Cardona et al., 2006). Furthermore, we recently reported that absence of CX3CR1 induces microglial-mediated IL-1β and neurotoxicity in the Akita model of DR (Cardona et al., 2015). Yet, as to what extent systemic inflammation influences the phenotype of retinal microglia remains elusive. Therefore, we stained for the proinflammatory markers IL-1β and inducible nitric oxide synthase (iNOS or NOS2, the enzyme required for nitric oxide synthesis), which are both recognized as key mediators that contribute to the pathogenesis of rodent and human DR (Tang and Kern, 2011). Nondiabetic 4 day LPS-treated CX3CR1-KO mice showed an increase expression of IL-1β in microglia (Figure 5A, yellow arrowheads), whereas minimal levels of IL-1β were detected in 4 day LPS-treated CX3CR1-HET mice (Figure 5A). Increased expression of IL-1β was also noted in cellular processes of GFAP+ astrocytes (Figures 5A,A_1_, yellow arrows). Similarly, most of the retinal microglia in the LPS-treated CX3CR1-KO retina colocalized to areas of iNOS immunoreactivity, and only a few NOS2^+^ activated microglia were observed in CX3CR1-HET tissues (Figure 5B, blue arrowheads). Indeed, qPCR analyses revealed that systemic inflammation induced a 2.6-fold increase of *Il1b* mRNA in the retinas of LPS-treated nondiabetic CX3CR1-KO compared to CX3CR1-WT mice (Figure 5C). Similarly, a 3.7-fold increase in *Il1b* and a 2-fold increase in *Nos2* relative expression was observed in LPS-treated Akita-KO compared to LPS-treated Akita-WT mice (Figures 5C,D). No significant differences were observed in the nondiabetic groups between *Nos2* expression, although the values were elevated relative to their respective naïve groups. Collectively, these data further bolster the concept that the absence of FKN signaling confers a proinflammatory and potentially toxic microglial phenotype that can perpetuate neuroinflammatory tissue damage.

### Fractalkine Dampens Retinal Microglia Activation, Perivascular Clustering and Fibrinogen Deposition in the Diabetic Retina Following Systemic Inflammation

**Figure 6:**
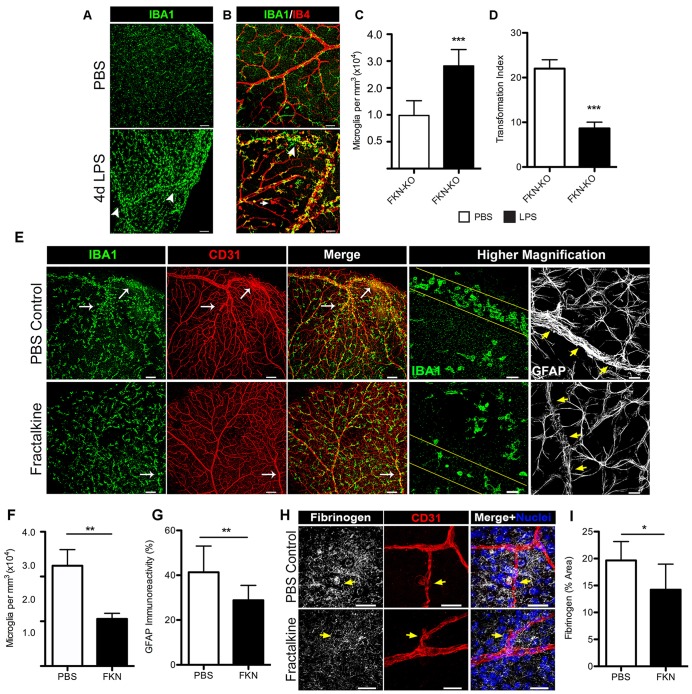
**Intravitreal (iv) treatment with recombinant fractalkine (FKN) dampens perivascular microglial clustering and fibrinogen deposition in diabetic mice after systemic inflammation. (A,B)** Confocal images of retinas from nondiabetic FKN-KO mice stained with **(A)** IBA1 (microglia, green) or along with **(B)** IB4 (blood vessels, red) following systemic inflammation (4 day LPS) and compared to naïve (mock injected with PBS) FKN-KO mice. White arrowheads indicate perivascular clusters of microglia in response to systemic LPS. Scale bars: **(A)** 100 μm, **(B)** 50 μm. **(C)** Microglial cells and **(D)** TIs indexes were quantified per image and data are presented as mean ± SD (*n* = 6; three images per mouse). **(E–I)** During 1 month of streptozotocin (STZ)-induced diabetes, acute systemic inflammation (2 day LPS) was induced in FKN-KO mice. Immediately following the second LPS injection, mice received an iv delivery of recombinant FKN (30 ng in 1 μL PBS) and an equal volume of PBS in the contralateral eye and retinal tissue were harvested and analyzed 24 h later. **(E)** Representative images of retinal tissue stained with IBA1 (microglia, green), CD31 (blood vessels, red) and GFAP (astrocytes, white) from diabetic FKN-KO mice treated with LPS and then either FKN or PBS administered into in the vitreous cavity. The higher magnification panels highlight less perivascular clustering of microglia and reduced GFAP staining around vessels in FKN treated tissues relative to controls (blood vessels outlined in either yellow lines or yellow arrows). Scale bars: 150 μm; higher magnification images are 30 μm. **(F,G)** Image quantification of **(F)** microglial and **(G)** GFAP immunoreactivity presents data as mean ± SD (*n* = 5; three images per mouse). **(H,I)** Confocal images of retinal tissue stained with fibrinogen (white), CD31 (red), Hoechst stain (nuclei, blue) and **(I)** fibrinogen immunoreactivity per image were quantified and expressed as percent area (*n* = 4, 2–3 images per mouse). Scale bars: 50 μm. ^*^P < 0.05, ^**^P < 0.01, ***P < 0.001 by Student’s *t*-test.

We next explored the scenario of utilizing recombinant FKN to reconstitute the retinal environment of FKN-KO mice in an attempt to attenuate microglial responses during systemic inflammation. For this, we first evaluated the phenotype of nondiabetic FKN-KO mice in response to 4 day LPS-induced inflammation, and found that the robust microglial activation and clustering phenotype observed in nondiabetic CX3CR1-KO and Akita-KO mice was replicated in the ligand deficient mice (Figures 6A–D). To evaluate the consequences of acute exposure of soluble FKN to a FKN void environment on regulating the observed microglial activation/clustering phenotype and extent of fibrinogen leakage in the diabetic retina, we established diabetes in FKN-KO mice via STZ administration. Diabetes was sustained in animals for 1 month to allow metabolic changes to normalize and to study acute hyperglycemia as similarly done in Akita mice. To limit the toxicity of STZ-induced diabetes in combination with systemic inflammation we only performed a 2 day LPS protocol in diabetic FKN-KO mice since we observed a similar trend (data not shown; Table 2) of increased microglial cellularity and fibrinogen leakage as observed in 4 day LPS-treated CX3CR1-KO mice (Figure 3). Intravitreal delivery of antibodies and steroids are currently approved methods for DR treatment, and thus we took advantage of that route of delivery to reconstitute the vitreous cavity for 24 h of diabetic FKN-KO mice following 2 day systemic inflammation in an attempt to rescue the observed phenotype. Mice that received sterile PBS (left eyes of mice serviced as vehicle controls) showed intense perivascular clustering of microglia and astrogliosis, in the context of GFAP staining, around blood vessels (Figure 6E). Whereas mice treated with recombinant FKN showed a reduction in perivascular microglia (Figure 6F) and GFAP staining (Figure 6G) in the retina. Indeed, robust fibrinogen staining was evident around disrupted vasculature in eyes that received PBS (Figure 6H, yellow arrows). In contrast a significant (*P* = 0.045) reduction in fibrinogen deposition was observed in FKN-treated eyes. Together, these data suggest that the soluble form of FKN can attenuate microglial activation during insult, and may also dampen inflammation providing an environment more conducive for repair.

## Discussion

It has been shown that fibrinogen leaks into the diabetic retina of patients when the BRB is compromised (Murata et al., 1992). In this study, we show that fibrinogen/fibrin deposition in the retina occurs as a result of chronic (16 weeks) but not acute (6 weeks) stages of diabetes, and is associated with activated microglia (Figure 1). Other investigators have shown that retinal vessels can become leaky early during diabetes in the context of detection of serum albumin within the retina. However, fibrinogen is a large glycoprotein with a molecular weight of 340-kDA relative to 65-kDA of albumin, highlighting the extent of barrier damage. Hyperglycemia increases the plasma levels of fibrinogen in patients (Murata et al., 1992; Schmidt et al., 1999; Duncan et al., 2003), due to inhibition of fibrinolysis, the enzymatic breakdown of fibrin (ogen) (Carr, 2001). Blood vessels may become more permeable/leaky as a consequence of early acute inflammation to promote extravasation of larger plasma proteins as in human DR (Asakawa et al., 2000) and thus accelerate inflammatory-mediated damage to the retina. This type of fibrinogen deposition was recapitulated in our mouse model. Although diabetes is a procoagulant prone disease, and endotoxemia has been shown to inhibit fibrinolysis in diabetic patients (Stegenga et al., 2008), knowledge regarding endotoxemia during early DR pathogenesis remains unknown. Interestingly, we found that during acute diabetes, an episode of endotoxin-induced systemic inflammation lead to early damage to the BRB and increased fibrinogen extravasation into the retina (Figure 2). This effect was significantly exacerbated in the absence of FKN signaling and fibrinogen deposition correlated with perivascular clustering of activated microglia in the diabetic retina (Figures 2–4).

During health, FKN/CX3CR1 signaling contributes to prenatal and postnatal brain development (Paolicelli et al., 2011; Hoshiko et al., 2012). Here, our results highlight the importance of the FKN/CX3CR1 signaling axis in retinal microglia function during DR and endotoxemia. Intriguingly, systemic LPS in nondiabetic animals resulted in fibrinogen leakage and robust microglial proliferation around areas of fibrinogen staining in CX3CR1-KO but not CX3CR1-HET mice. Histochemical analyses revealed that systemic LPS upregulated IL-1β levels in perivascular microglia and astrocytes (Figure 5). Interestingly, IL-1β is known to promote vascular leakage and angiogenesis. Whereas, blocking IL-1β improved endothelial dysfunction in diabetic models (Vallejo et al., 2014) and prevented choroidal neovascularization in models of laser-induced retinal degeneration (Lavalette et al., 2011). Indeed, both microglia (Halle et al., 2008) and astrocytes (Minkiewicz et al., 2013) have the machinery to release bioactive IL-1β levels. Also, activated microglia release ATP (Pascual et al., 2012) increasing extracellular danger signals that in an autocrine and paracrine fashion may sustain microglial and astrocytic release of IL-1β in the retina. Along those lines, CNS endothelial cells express interleukin-1 receptor type 1 (IL-1R1) and have been recently shown to respond to myeloid derived IL-1β and contribute to neuroinflammation and autoimmune disease (Lévesque et al., 2016). We speculate based on our results that CX3CR1 contributes to maintaining the integrity of the BRB during endotoxemia, perhaps indirectly by repressing proinflammatory cytokines released from activated microglia and astrocytes that can act on endothelial cells. Lastly, interaction of FKN with fibrinogen directly at the vascular site may dampen the influx of fibrinogen and hence hindering microglia activation. These latter hypotheses need further investigation. However, the results provide observations that are consistent with the spatial correlation of fibrinogen deposition and microglia activation as reported in animal models of multiple sclerosis (MS; Akassoglou et al., 2004; Adams et al., 2007; Davalos et al., 2012) and Alzheimer’s disease (AD; Paul et al., 2007).

This study suggests a novel working model for the pathology of DR with direct implications in microglial lesion formation, disruption of vascular integrity, and perivascular fibrinogen deposition in the diabetic retina. Fibrinogen is a plasma protein that under normal homeostatic conditions does not reach the brain, spinal cord or retinal tissues. However, leaky vessels allow the extravasation of fibrinogen and exposure to neuronal tissues where is deposited as insoluble fibrin (Davalos et al., 2012). The inflammatory effects of fibrinogen are associated with neurological diseases such as MS (Adams et al., 2007; Davalos et al., 2012; Ryu et al., 2015), stroke (Cheung et al., 2008) and AD (Flemming, 2014). Importantly, fibrinogen has been linked to microglial activation *in vitro* and *in vivo*, and a causal relationship between fibrinogen deposition and perivascular clustering of microglia in the CNS in experimental models of neuroinflammation has been described (Davalos et al., 2012; Ryu et al., 2015). Indeed, depletion of fibrinogen or inhibition of fibrin formation by anti-coagulants suppressed microglial activation and disease pathogenesis in animal models of MS (Adams et al., 2007; Han et al., 2008; Davalos et al., 2012). This report here only highlights the relevance of fibrinogen as a marker of vascular leakage and association with microglial activation. Future studies will explore the potential role of fibrinogen leakage into the retina. Moreover, characterization of the type of vascular degeneration in response to systemic inflammation, such as quantification of acellular, nonperfused capillaries and pericyte dropout would help elucidate the extent of vasoregression and the potential occurrence of neovascularization.

Intravitreal injection injection has become the most accepted ophthalmic procedure in the treatment of DR, and in particular, for the administration of anti-VEGF agents in neovascular age-related macular degeneration, and other various vascular disorders (McCannel, 2011). These therapeutic alternatives are mostly implemented at chronic stages of disease (Cheung et al., 2010) and efforts to treat early DR are hindered by the lack of mechanistic insights on disease initiation and progression. Our study reveals a novel outcome of soluble FKN in the treatment of DR. Introducing soluble FKN into the retinas of diabetic (STZ) FKN-KO mice was sufficient to attenuate microglial perivascular clustering and reduce fibrinogen deposition during acute endotoxin-induced inflammation (Figure 6). Accordingly, it has been demonstrated that increasing FKN/CX3CR1 signaling via iv delivery of FKN, dampens microglial activation and photoreceptor degeneration in a model of retinal disease (Zabel et al., 2016). Therefore, future studies addressing the effect of FKN administration in visual acuity will be valuable to validate its use to ameliorate retinal damage during retinopathy. As discussed earlier, potential interactions between microglia and astrocytes will be valuable to dissect, since astrogliosis, in the context of GFAP staining (which notably also stains Müller cells in the retina), was also diminished near blood vessels in FKN treated eyes (Figures 6E,G). Astrocytes along with Müller cells constitute a major glial cell population in the retina and provide neuronal, vasculature and vision support. Evidence supports that astrocytes and Müller cells provide protective functions during diabetes, however, these cells have the capacity to release proinflammatory mediators and contribute to oxidative stress during DR (Xu et al., 2014). Thus, the concomitant regulation of reducing the proinflammatory release of cytokines in microglia and macroglia may yield promising therapeutics for DR treatment. Given the decrease of FKN in retinal tissues of Akita-WT mice (Cardona et al., 2015), the potential benefits of FKN administration presents a promising scenario for DR and other retinal diseases. Furthermore, the research presented here poses important clinical relevance for humans carrying the polymorphic variant *CX3CR1*^I249/M280^ (*CX3CR1*^M280^) estimated in about 20% of the population. These changes in human CX3CR1 decrease FKN affinity (McDermott et al., 2003) and several studies support a role for *CX3CR1*^M280^ in susceptibility to age-related macular degeneration (Tuo et al., 2004; Chan et al., 2005; Brión et al., 2011; Schaumberg et al., 2014). Together, our data suggest a pivotal role for FKN/CX3CR1 signaling in governing microglial responses during insult, and thus modulating CX3CR1 signaling may be relevant alternative approaches to mitigate tissue pathology in the diabetic retina.

## Author Contributions

ASM performed research and analyzed data; RG, SMC and SAM performed research; KA interpreted data and designed data analysis, AEC conceived the study, designed experiments, analyzed data. ASM, SAL, KA and AEC wrote the article. All authors read and approved the final manuscript.

## Funding

This study was supported in part by funds from the San Antonio Area Foundation (Grant 201135345 to AEC), The National Institutes of Health (SC1GM095426 and R01 NS078501 to AEC, and R35 NS097976 to KA), The National Institute on Minority Health and Health Disparities (RCMI Grant G12MD007591), and the UTSA RISE-PhD Trainee Program (Grant GM060655 to ASM).

## Conflict of Interest Statement

The authors declare that the research was conducted in the absence of any commercial or financial relationships that could be construed as a potential conflict of interest.
